# The responses of poplars to fungal pathogens: A review of the defensive pathway

**DOI:** 10.3389/fpls.2023.1107583

**Published:** 2023-02-16

**Authors:** Yi Zeng, Haifeng Song, Linchao Xia, Le Yang, Sheng Zhang

**Affiliations:** ^1^ Key Laboratory of Bio-Resource and Eco-Environment of Ministry of Education, College of Life Sciences, Sichuan University, Chengdu, China; ^2^ College of Grassland, Resources and Environment, Inner Mongolia Agricultural University, Hohhot, China

**Keywords:** poplar, fungus, physiology, molecular mechanism, defense

## Abstract

Long-lived tree species need to cope with changing environments and pathogens during their lifetime. Fungal diseases cause damage to trees growth and forest nurseries. As model system for woody plants, poplars are also hosts of a large variety of fungus. The defense strategies to fungus are generally associated with the type of fungus, therefore, the defense strategies of poplar against necrotrophic and biotrophic fungus are different. Poplars initiate constitutive defenses and induced defenses based on recognition of the fungus, hormone signaling network cascades, activation of defense-related genes and transcription factors and production of phytochemicals. The means of sensing fungus invasion in poplars are similar with herbs, both of which are mediated by receptor proteins and resistance (R) proteins, leading to pattern-triggered immunity (PTI) and effector-triggered immunity (ETI), but poplars have evolved some unique defense mechanisms compared with *Arabidopsis* due to their longevity. In this paper, current researches on poplar defensive responses to necrotrophic and biotrophic fungus, which mainly include the physiological and genetic aspects, and the role of noncoding RNA (ncRNA) in fungal resistance are reviewed. This review also provides strategies to enhance poplar disease resistance and some new insights into future research directions.

## Introduction

Forest trees play crucial roles in mitigating effects of climate change and increasing industrial demand, and have considerable economic and ecological value (Kovalchuk et al., 2013). Poplars are predominantly distributed worldwide as model species of woody plants due to their rapid growth and stress tolerance. *Populus* is also hosts of a large variety of fungus (Miranda et al., 2007; Wang et al., 2017a; Wang et al., 2022). Fungal diseases not only affect the growth, but also cause large numbers of tree deaths and ecosystem degradation (Eyles et al., 2010; Kovalchuk et al., 2013). This makes it ecologically and economically important to deepen the knowledge of poplar defense mechanisms against fungus (Kovalchuk et al., 2013). Plant pathogens can be broadly divided into biotrophic (feeding on living plant tissue), necrotrophic (feeding on dead plant tissue) and hemibiotrophic (infect living plant tissues to first establish infection before switching to necrotrophy) (McCombe et al., 2022). Biotrophs infecting poplars like leaf rust, caused by obligate parasitic fungus *Melampsora* spp., powdery mildews caused by *Phyllactinia* spp. or *Uncinula* spp., while necrotrophs or hemibiotrophs, like leaf blight (caused by *Septoria* spp.), leaf spot (caused by *Marssonina* spp., *Venturia* spp.*, Coryneum* spp.), canker (*Septoria* spp), and so on (Steenackers et al., 1996; Weiland et al., 2003; Feau et al., 2010). In order to improve the resistance of trees to disease, it is necessary to understand the defense mechanisms.

**Figure 1:**
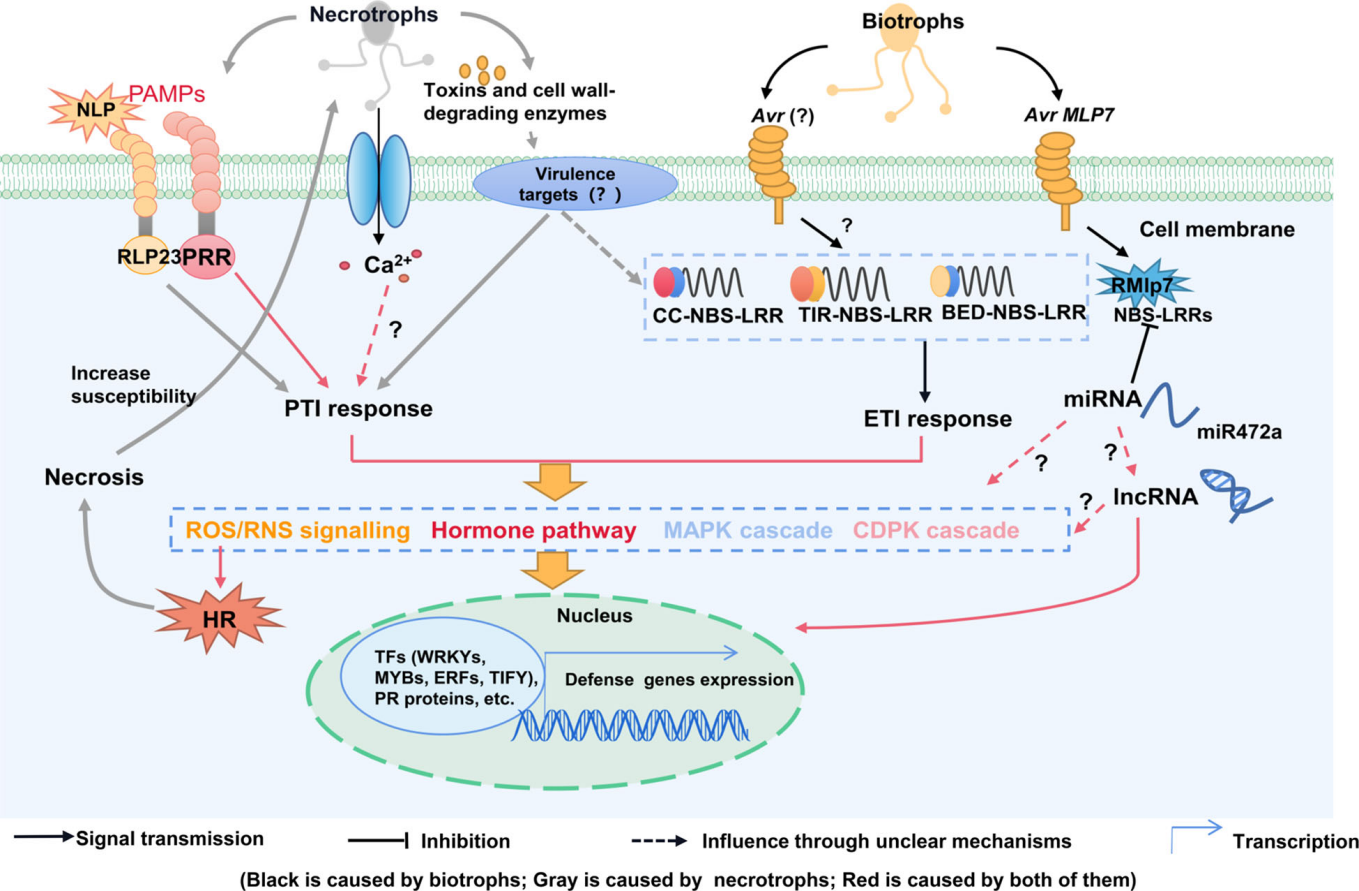
A model of poplars immunity to fungal pathogens. When plants attacked by biotrophic pathogens, PRRs recognize extracellular pathogenic characteristics and activate PTI, R proteins (NBS-LRRs) recognize intracellular effector proteins from pathogens and activate ETI. They induce a series of defense responses including the production of ROS, RNS, phytohormone, CDPKs and MAPK signals. But necrotrophic fungus may only recognized by PRRs. Many defensive genes and transcription factors are key players, including *PR*, MYB, WRKY, TIFY, and ERF, lncRNAs and miRNAs also participate in defense responses against fungal pathogens. miRNAs also play a role in the regulation of NBS-LRR. *Avr*, *avirulence gene*; CDPK, calcium-dependent protein kinase; ETI, effector-triggered immunity; HR, hypersensitive response; lncRNAs, long ncRNAs; MAPK, mitogen-activated protein kinase; miRNAs, microRNAs; PCD, programmed cell death; PR, pathogenesis-related proteins; PRRs, pattern recognition receptors; PTI, pattern-triggered immunity; ROS, reactive oxygen species.

Similar with herbaceous plants, poplars defense mechanism categorized as constitutive defenses and induced defenses. Induced defenses only expressed when plants suffered external stimulus, and constitutive defenses are always expressed in the plants (Eyles et al., 2010). Constitutive defense is the first line of defense contributing to non-host resistance, including inherent physical structures and phytochemicals, which provide basic defense against pathogens (Alkan and Fortes, 2015). To reduce morbidity during long life cycle of poplar, they enhance the physical and phytochemical defenses. Furthermore, plants enhance defensive capacity through a series of complex regulations when infected by pathogens, which is called induced defenses. According to the expression range, induced resistance can be divided into local induced resistance and systemic induced resistance (De Kesel et al., 2021). Trees evolve induced defenses due to the lower resource allocation costs than constitutive defenses (Eyles et al., 2010). Regardless of the lifestyle of the attacking pathogens, plants have developed several means in protection against fungal pathogens: pathogen-associated molecular pattern PAMP-triggered immunity (PTI), effector-triggered immunity (ETI) and noncoding RNA (ncRNA)-mediated defense (De Kesel et al., 2021) (**Figure 1**). To against fungal pathogens, plants need to distinguish different fungal life cycles, for example, programmed cell-death (PCD) around the infected sites is an effective way against biotrophs but not an appropriate response to some necrotrophy.

**Figure 2:**
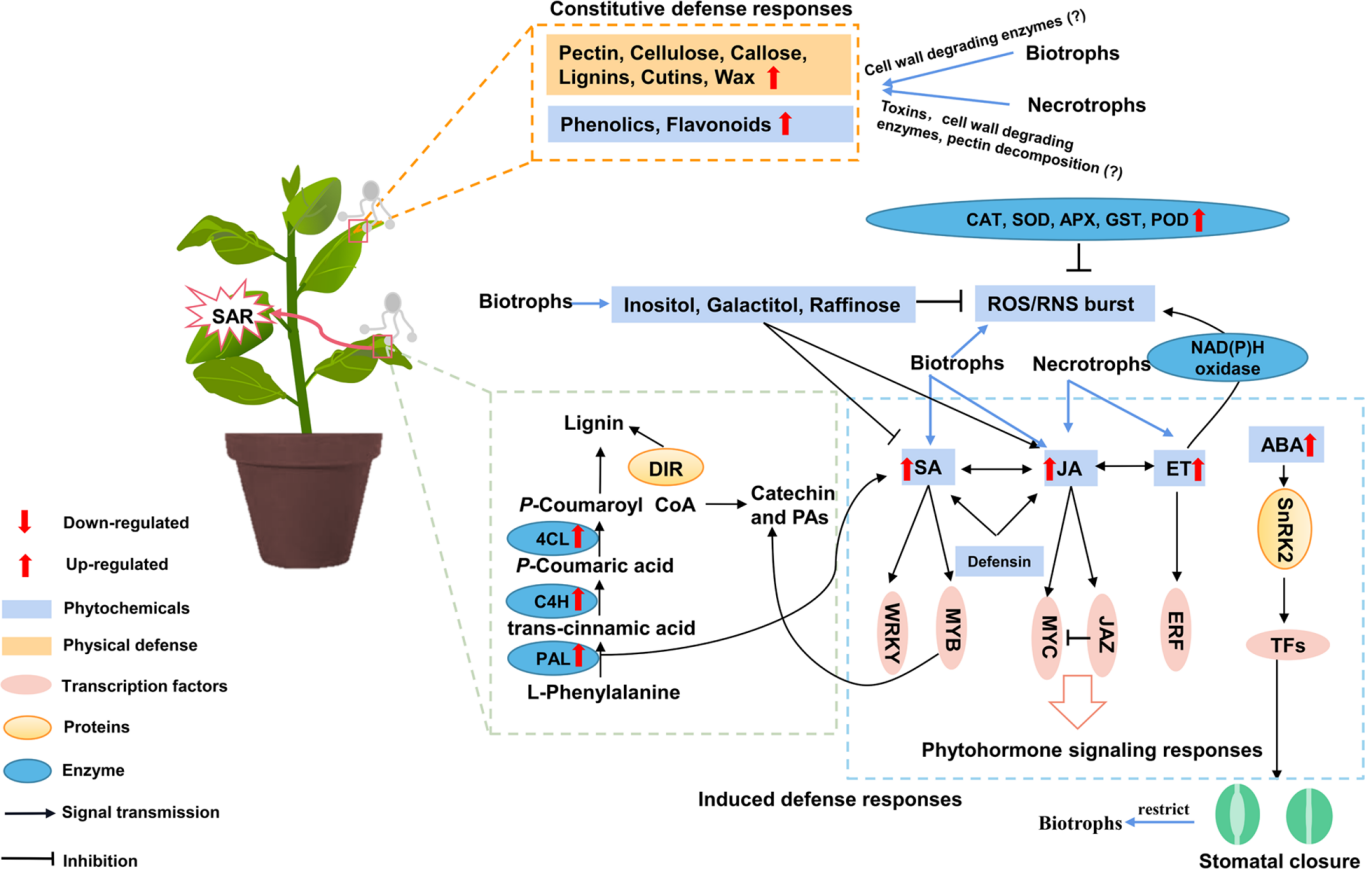
A model for the interaction between poplar physical and biochemical defenses. Constitutive defense responses mainly include physical barriers and phytochemicals defenses, when attacked by pathogens. Defense-related metabolites include peroxide, phenolics and phytohormones. The black arrows represent responses caused by biotrophic fungus, grey arrows represent responses caused by necrotrophic fungus, red arrows represent responses caused by all fungus. ABA, abscisic acid; APX, ascorbate peroxidase; C4H, cinnamate 4-hydroxylase; CAT, catalase; 4CL, 4-coumarate-CoA ligase; DIR, dirigent; ET, ethylene; GST, glutathione sulfur transferase; JA, jasmonic acid; JAZ, jasmonate-zim domain; PAL, phenylalanine ammonia lyase; PAs, proanthocyanidins; POD, peroxidase; ROS, reactive oxygen species; SA, salicylic acid; SOD, superoxide dismutase; SAR, systemic acquired resistance.

The completion of the whole genome of *P. trichocarpa* marks that the study of poplar disease has entered the genomic era (Tuskan et al., 2006). However, it is difficult to study the disease resistance of poplars using genetic methods due to the long generation time. To date, research on poplar-fungus interactions at the molecular level has mostly focused on genes related to host defense. Therefore, this paper mainly summarizes the research on physical and physiological mechanisms in poplars against fungus with different life cycles (**Figure 2**). Also, we have focused on the molecular mechanism especially on defense genes, transcription factors and non-coding RNAs in poplars against biotrophic and necrotrophic fungus (**Tables 1**, **2**).

**Table 1:** The genes involved in poplars defense response to biotrophic fungus.

Genes	Functions in disease resistance	pathogens	References
*PsRPM1*	Contain NBS-LRR domains, while miRNAs are down-regulated. miRNAs negatively regulate PsRPS2/5 and *PsRPM1* genes to enhance the resistance of poplars to rust fungus	*M. larici-populina*	Liu et al., 2019
*PsRPS2/5*			
*EDS1*	Activates the TIR domain of R proteins and acts upstream of SA to regulate SA accumulation		Chen et al., 2021
*NDR1*	Activates the CC domain-containing R proteins and acts upstream of SA to regulate SA accumulation		
*RISP*	Play important roles in early defense against rust		Petre et al., 2016
*MAPK*	Participate in downstream signal transduction by phosphorylation	*M. medusae*	Hamel et al., 2005
*MAPKKK5*		*M. larici-populina* *M. medusae*	Azaiez et al., 2009
*CDPKs*			
*GST*	Encodes enzymes of the redox regulation pathway	*M. larici-populina*	Rinaldi et al., 2007
*GOLS3*	Participates in galactose and raffinose synthesis to mitigate the defense response to pathogens through suppressing ROS	*M. aecidiodes*	La Mantia et al., 2018
*CsRFS*			
*PIP5K*	Leads to accumulation of raffinose		La Mantia et al., 2013
*PtrMYB134*	anthocyanin and proanthocyanidin biosynthesis	*M. medusae*	Mellway et al., 2009
*R2R3MYB*	Activates flavan-3-ol biosynthesis	*M. larici-populina*	Ullah et al., 2019a
*PtrWRKY23*	Disrupts redox homeostasis and cell wall metabolism	*M. medusae*	Levee et al., 2009
*PtrWRKY89*	Promotes the expression of *PR* gene	*M. larici-populina*	Jiang et al., 2017
*PtrWRKY18*	Potential target genes of *WRKY89*		
*PtrWRKY35*			
*PR1*	Relates to the abundance of the rust pathogen	*M. larici-populina*	Ullah et al., 2022
*Chit* (*chitinase gene*)	a class of PR proteins	*M. medusae*	Miranda et al., 2007
*KTI*	Activates or inhibits the formation of PCD	*M. larici-populina*	Foster et al., 2015 Chen et al., 2021
*CYP*	Plays an important role in both the JA and SA pathways	*M. larici-populina*	

**Table 2:** The genes involved in poplars defense response to necrotrophic or hemibiotrophic fungus.

Genes		Functions in disease resistance	pathogens	References
*MAPK*		Participate in downstream signal transduction by phosphorylation	*S. musiva*	Hamel et al., 2005
*MPK3*		Participate in ET signaling	*M. brunnea* (*Marssonina*)(hemibiotrophs)	Zhang et al., 2018
*MPKK9*				
*PtoMYB115*		Activates anthocyanin and proanthocyanidin biosynthesis	*D. gregaria*	Wang et al., 2017a
*PsnWRKY70*		Activates MAPK cascade genes, calcium ion signal-related genes, membrane receptors, other members of WRKYs, and LRR domain proteins LRR8, LRR-RLK, ADR1-like 2, NB-ARC, etc.	*A. alternata*	Wang et al., 2022
*PtrWRKY40*		Negatively regulate SA-related genes expression, but increase resistance to necrotrophic fungus in *Arabidopsis* by activating JA-related genes expression	*D. gregaria*	Karim et al., 2015
*PeTLP*		a class of PR proteins	*M. brunnea* (*Marssonina*)(hemibiotrophs)	Sun et al., 2020
*Chit* (*chitinase gene*)		a class of PR proteins	*S. musiva* *A. alternata*	Liang et al., 2014 Huang et al., 2022
*KTI*		Activates or inhibits the formation of PCD	*S.musiva* *S. Populicola*	Foster et al., 2015 Chen et al., 2021
*PtDIR11*		Involves in process of plant lignin synthesis	*S. populiperda*	Li et al., 2022
*PtDefensin*		Involves in JA and SA signalling and increased H_2_O_2_ levels	*S. populiperda*	Wei et al., 2020a Wei et al., 2020b
*MsrA2*		Host defense peptide (HDP)	*S. musiva*	Yevtushenko and Misra, 2019
*miR472a*		miR472a negatively regulated NBS-LRRs, leading to a ROS burst and HR, which increase susceptibility to necrotrophic fungus the, but enhance defense response to hemibiotrophic fungus	*C. chrysosperma* *C. gloeosporioides* (hemibiotrophs)	Su et al., 2018

## Defense responses of poplars to fungal pathogens

### Constitutive defense responses

Biotrophic fungal pathogens such as rust and powdery mildew produce invasive filaments through appressorium, and penetrate the cuticle, and form haustoria in the epidermal cells to absorb nutrients from the hosts, and produce cell wall degrading enzymes and sporulate without killing the host cells (Maupetit et al., 2018). Whereas most necrotrophic fungi kill their hosts by secreting cell wall-degrading enzymes or toxins to impair cuticles and cell walls to facilitate infection (Shi et al., 2016). Biotrophic fungal pathogens spend most of their life cycle on living plant tissues, therefore, their fitness appeared to be more influenced by constitutive defense (Maupetit et al., 2018). Constitutive defense is the first line of protection, including physical structures and phytochemicals. Woody plants have many mechanical barriers to against pathogen invasion, such as the leaf cuticle, the pectin and lignin of cell walls. Cuticle and cell wall are the first line of defense in plants, the components of cell wall are cellulose, hemicellulose, pectin, and lignin (Ziv et al., 2018). Many biotrophic fungal species use their appressoria to penetrate the cuticular layer and then infect internal cells. The cuticle is a polyester that is partly covered with waxes (epicuticular and intracuticular) (Serrano et al., 2014). A study found *PtoMYB142* could directly regulate the transcriptional activity of wax biosynthesis genes, *e.g*., fatty acid hydroxylase (*CER4*) and 3-ketoacyl CoA synthase (*KCS6*), to adapt drought conditions for poplars (Song et al., 2022). But the contribution of wax biosynthesis on poplar disease resistance is poorly studied. However, many rust fungi failed to penetrate the cuticle and thus had to invade the mesophyll cells through the stomata by germ tubes, hyphae or appressorium (Rinaldi et al., 2007), but the cuticle was also found to contribute to non-host resistance to leaf rust by impeding the germination and growth of urediniospores from *M. larici-populina* (Yu et al., 2019). Lignin also acts as physical barrier during pathogen infection, preventing water and nutrients transferring from host cells to pathogens (Ellinger et al., 2013; Miedes et al., 2014). Genes involved in lignin biosynthesis are critical for plant cell walls in immunity, *e.g*., *phenylalanine ammonia lyase* (*PAL*), *cinnamate 4-hydroxylase* (*C4H*), *4-coumarate-CoA ligase* (*4CL*), *cinnamyl alcohol dehydrogenase* (*CAD*), *cinnamoyl-CoA reductase* (*CCR*) and *hydroxycinnamoyl transferase* (*HCT*), were highly expressed after attack by fungi pathogens (Azaiez et al., 2009; Polle et al., 2013; Huang et al., 2022). Interestingly, in incompatible interaction, localized lignin formation was observed, while there was an accumulation in compatible interaction when poplars infected with *Melampsora* (Boyle et al., 2010). In the incompatible interactions, abundant lignin was deposited around leaf vessels (Rinaldi et al., 2007). Regulation of the lignification pathway may be critical for improved poplar tolerance (Polle et al., 2013). Dirigent (DIR) proteins have been identified in many plants, which are involved in process of plant lignin synthesis. Furthermore, overexpression of *PtDIR11* in poplars could improve lignin biosynthesis and enhance poplar resistance to *Septotis populiperda* (Li et al., 2022). These results imply that lignins are effective antifungal chemical defenses against pathogens infection.

Some necrotrophic fungus prefer to plants with cell walls are rich in pectins because they possess a strong pectin decomposition machinery. Pectin methylesterase (PME), a class of pectin modification enzyme, plays an important role in cell wall modification, pectins function not only in primary cell walls, but also in secondary cell walls. It is an effective way in restricting necrotrophic fungal pathogens to inhibit PME (Pelloux et al., 2007). It has been reported *PtoPME35* involved in stomatal closure, and the overexpression of its homologous gene *AtPME35* in *Arabidopsis* leading to plant lodging, but overexpression of *PtoPME35* do not influence poplar growth, indicating that woody plants have more complex defensive networks than annual herbs (Yang et al., 2020). However, cell wall-related genes showed differential expression patterns when two susceptible poplars infected by hemibiotrophic pathogen of *M. brunnea*. For example, pectin methylesterase inhibitors were enriched in *P. deltoids* but they were mostly reduced in *P. alba*. Transcriptomic data showed that *P. deltoids* differentially expressed genes were most responsive at the initial biotrophic stage, while *P. alba* was mainly responsive to *Marssonina brunnea* at the necrotrophic phase (Zhang et al., 2018). Additionally, when susceptible poplars suffered two specific forms of *M. brunnea*, the number of DEGs expressed among three stages of infection were changed in a significantly different pattern. The results showed there were more differentially expressed genes in the necrotrophic stage than biotrophic stage when poplars infected with *M. brunnea*. In particular, pectinlyase was significantly induced in multi-strain infections during the initial invasion phase, but not expressed in monoculture infections (Ren et al., 2020).

### Induced defense responses

#### Innate immunity against fungal pathogens

Similar with herbs, woody plants sense pathogen invasion through resistant (R) proteins or receptor proteins (Kovalchuk et al., 2013). When fungal pathogens penetrate plant physical barriers, PRRs may recognize pathogen-associated molecular patterns (PAMPs) from pathogens and activate PTI. As shown in **Figure 1**, PTI acts as a basic defense system and triggers a set of plant defense responses, including activation of signaling molecules, like calcium-dependent protein kinase (CDPK) and mitogen-activated protein kinase (MAPK) cascades. CDPKs and MAPK are involved in the regulation of downstream immune responses (Meng and Zhang, 2013). In poplars, MAPK has been reported to be an important component in biotic and abiotic stresses, and functioned as common points of cross-talk between pathogen defense and oxidant stress signaling cascades (Hamel et al., 2005). A study indicated that genes encoding mitogen-activated protein kinase-kinase-kinase 5 (MAPKKK5) and CDPKs were upregulated after infection by rust (Azaiez et al., 2009). When two susceptible poplars infected with *M. brunnea* (*Marssonina*) that causes leaf spot disease, *MPK3* and *MPKK9* were detected in both poplars (Zhang et al., 2018), and the *MPKK9-MPK3* modules have been identified to participated in ethylene (ET) signaling (Yoo and Sheen, 2008). However, the specific functional mechanisms of MAPK and CDPK regulating poplars defense against pathogens remains to be further studied. The PAMP flg22 is a highly conserved 22-amino-acid peptide of the N-terminal of bacterial flagellin, which can induce PTI responses in many plants (Zhao and Cheng, 2022). The activation of MAPK cascades after flg22 treatment was found in *P. davidiana* × *P. bollean*a, but necrosis and ethylene-inducing peptide 1-like proteins (NLPs), which mainly expressed in hemibiotrophs or necrotrophs, cannot generate PTI responses in general. Heterologously expressed receptor-like protein (*AtRLP23*) in poplar recognizes NLP24s and generates PTI responses and ROS bursts after *M. brunnea* and *Elsinoë australis* infections, thereby enhancing broad-spectrum disease resistance to the fungus (Zhao and Cheng, 2022) (**Figure 1**). Pathogens have evolved effectors that promote their growth by suppressing PTI, which results in effector-triggered susceptibility (ETS). However, plants will evolve *R* gene to sense the effectors and trigger ETI, which is associated with the hypersensitive response (HR) (Chisholm et al., 2006). PTI and ETI reinforcing each other to enhance plant defensive responses against pathogen infections by inducing downstream signal transduction (Chang et al., 2022). However, the crosstalk of PTI and ETI in poplar still needs to be studied.

Plants recognition events are mostly mediated by a class of receptor proteins containing nucleotide-binding (NB) and leucine-rich repeat proteins (LRR) domains (Dodds and Rathjen, 2010). The NBS-LRR class is the most abundant R protein and is responsible for pathogen identification of intracellular effectors (Dodds and Rathjen, 2010). R-mediated defense response seem not to function in necrotrophs, while PRRs like receptor-like kinases (RLKs) involved in the perception of necrotrophs (Wang et al., 2014), but R-mediated plant cell necrosis increased susceptibility to necrotrophs. Therefore, R protein acts indirectly on necrotrophic fungus (Su et al., 2018) (**Figure 1**). In dicotyledons, there are two main classes of *NBS-LRRs*: *TIR-NBS-LRRs* and *CC-NBS-LRRs*, which have Toll-interleukin-1 receptor (TIR) and amino-terminal coiled-coil (CC) domains, respectively (Bresson et al., 2011). TIR-NBS-LRRs are entirely missing from the monocotyledon’s genome like rice. In poplars, a third class of *NBS-LRR* genes, called *BED-NB-LRRs*, which containing a BED domain, has been reported. BED-NB-LRR family comprising 32 members, seems to be unique to poplars (Kohler et al., 2008; Germain and Seguin, 2011). Poplar possesses 400 *NBS-LRRs* nearly twofold that of *Arabidopsis*, indicating that woody plants may have developed more intracellular receptors compared with herbaceous plants due to their longevity, which may result in greater disease resistance ability (Duplessis et al., 2009). There were 34 *NBS-LRRs* differentially expressed after rust fungi infected poplars (Kohler et al., 2008). It has been reported disease resistance proteins (CC-NBS-LRR class) and LRR proteins were induced by biotrophic fungus in hybrid poplars (Azaiez et al., 2009). In addition, enhanced disease susceptibility 1 (EDS1) and nonrace-specific disease resistance 1 (NDR1) are required for activation of R protein-mediated resistance. *EDS1* regulates defense signaling by activating R proteins with TIR domains, while *NDR1* is required for the activation of CC domain-containing R proteins (Lang et al., 2022). *EDS1* and *NDR1* were found to regulate SA accumulation in poplars in response to leaf rust disease (Chen et al., 2021). Rust-induced secreted protein (RISP) is a small (82 amino acids), cysteine-rich protein and was highly inducible in poplar leaves after infection with rust, and *RISP* was found near the *LRR-RLP* gene (Petre et al., 2014). The two genes have similar promoter regions and expression profiles in response to rust infection. RISP inhibits *M. larici-populina* growth on poplar leaves by binding to *M. larici-populina* urediniospores and inhibiting germination and germ tube elongation. Thus, *RISP* plays a role in early defense against fungal pathogens (Petre et al., 2016) (**Table 1**).

In the plant-pathogen interaction system, pathogen-induced host-specific resistance mainly depends on pathogen *avirulence* (*Avr*) genes and *R* genes to activate downstream defense cascades, forming a HR at the infected site (Gururani et al., 2012). However, as *Avr* evolves rapidly, it allows pathogens to break down *R*-mediated plant immunity. For example, the *AvrL567*, a virulence gene, was found in *M. lini* haustoria and induced by HR (Dodds et al., 2004). The transcriptome data indicated that six known *Avr* genes in *M. lini* (*AvrM*, *AvrM14*, *AvrL2*, *AvrL567*, *AvrP123* and *AvrP4*) showed similar patterns of early expression during infection (Wu et al., 2019). However, the *R* genes that sense these *AVR* genes in poplars are still unknown. Another study showed that the candidate locus *AvrMlp7* drives rust fungus adaptation to poplar *RMlp7*-mediated immunity (**Figure 1**) (Louet et al., 2021).

#### HR and ROS inhibit fungus infection

HR is the most typical response after infection and causes programmed cell death (PCD). HR has been extensively described with relation to biotrophic fungi, but promote necrotrophic pathogens infection (Mayer et al., 2001). A study found that several genes in poplars that cause HR were upregulated at 96 hpi (necrotrophic phase) when response to *M. brunnea*, indicating that necrotrophic fungus may induce HR in poplars to increase their susceptibility (Zhang et al., 2018). HR varies among different poplar-pathogen interaction types. In incompatible *Populus*-*Melampsora* interactions, HR appears the day after infection, and poplars then show PCD characteristics at 7 dpi, however, there is no HR performance in the compatible interaction (Rinaldi et al., 2007). One of the most striking features accompanying HR is the burst of ROS after pathogen infection. ROS burst is one of the earliest responses of plants to fungus, leading to necrosis of host tissue, which develop an effective defense against biotrophic fungi but may increase susceptibility to necrotrophs (Mayer et al., 2001). Therefore, ROS homeostasis regulation is important for regulating plant resistance to different pathogens.

Plants have initiated a series of enzymatic and nonenzymatic oxidation systems to reduce ROS accumulation and to avoid oxidative damage in cells. Several transcripts encoding ascorbate peroxidase (APX), superoxide dismutase (SOD), glutathione sulfur transferase (GST) and peroxidase (POD) were upregulated (**Figure 2**), which were involved in the oxidative burst and even reached approximately 20-fold higher levels in *M. larici-populina* and *M. medusae* infected poplars (Miranda et al., 2007; Azaiez et al., 2009). However, the accumulation of ROS is varied from different poplar species. A study on male and female *P. cathayana* infected with rust showed that the production of ${\text{O}}_{2}^{-}$ was higher in males than in females, while the H_2_O_2_ content was higher in females than in males. The results indicated that male poplars showed higher antioxidant activities and less H_2_O_2_ accumulation than females after being infected by leaf rust; therefore, rust disease was more severe in female poplars (Zhang et al., 2010). In addition, when poplars are attacked by pathogens, guard cells produce H_2_O_2_ to form a rapid defense, but it is weaker in the compatible interactions (Boyle et al., 2010). Moreover, second messengers, *e.g*., Ca^2+^, hydrogen sulfide (H_2_S), inositol triphosphate (IP3) and NO, are produced within seconds to enhance plant responses (Agurla et al., 2018). For example, NO-activated antioxidant enzymes can decrease ROS and reactive nitrogen species (RNS) toxicity to improve poplar tolerance to environment stress (Cheng et al., 2016). However, how these second messengers regulate biotic stress in poplars remains unclear. Additionally, inositol, galactitol and raffinose are important regulators of ROS homeostasis. Inositol negatively impacted SA, while galactitol enhances systemic resistance to necrotic pathogens induced by JA (**Figure 2**). The overexpression of galactose synthase gene (*GOLS3*) and raffinose synthase gene (*CsRFS*) mitigated the defensive responses to poplar leaf rust by suppressing ROS and attenuating calcium and phosphatidic acid signaling events. The accumulation of galactinol could constitutively repress defense signaling events upstream of SA biosynthesis (La Mantia et al., 2018). *Phosphatidylinositol 4-phosphate 5-kinase* (*PIP5K)* encoding phosphatidylinositol 4-phosphate 5-kinase led to the accumulation of raffinose. In *P. trichocarpa* × *deltoides*, the expression of *PIP5K* decreased after 48 hpi inoculation with rust (La Mantia et al., 2013). WRKY-TF also plays an important role in multiple defense responses (Spoel and Loake, 2011). For example, *WRKY23* affects poplar resistance to fungi infection by disrupting redox homeostasis and cell wall metabolism. *WRKY23* can increase poplar susceptibility to rust disease (Levee et al., 2009). Thus, precise regulation of the transcription factors and genes related to ROS production could improve the resistance of poplars to pathogens (**Figure 2**).

#### SA-mediated signaling pathway against pathogens

Phytohormones play essential roles in plant resistance to pathogens and plant immune responses (Pieterse et al., 2012; Chanclud and Morel, 2016). SA dominates in the execution of host defense response against biotrophic, while JA and ET are key players to facilitate host defense response against necrotrophic (Li et al., 2019). In plants, SA is synthesized through two routes. One route is the chloroplast-localized isochorismate synthase (ICS) pathway, and the other is PAL-mediated pathway. ICS pathway is believed to be responsible for the most of SA synthesized during the activation of pathogens in *Arabidopsis* (Yuan et al., 2009). While *Populus* is primarily dependent on the PAL pathway, therefore, *PAL* genes are critical for SA synthesis. SA might be converted to MeSA at infected sites and transported as a signaling molecule to uninfected sites, induced SAR (Li et al., 2018). SA signaling positively regulates plant defense against biotrophic pathogens by enhancing secondary metabolites and inducing *pathogenesis-related genes* (*PRs*), which are necessary for the establishment of SAR (Shah, 2003; Thaler et al., 2012). SA was proved to activate flavan-3-ol biosynthesis against biotrophic fungus *M. larici-populina* in poplars (Ullah et al., 2019a). In addition, it was indicated that the accumulation of flavanols was negatively regulated by cytokinin (CK) in poplars (Ullah et al., 2019b).

High SA levels induce *PR* gene expression in *Arabidopsis*, which may lead to high metabolic costs. However, for perennial woody plants, it may have better balance of resource allocation, therefore, *PR* gene expression is not necessarily with SA levels, but positively correlate with the degree of disease susceptibility in poplars (Ullah et al., 2022). Evidence has shown that an increase of *PR* gene expression after pathogens invasion, is probably mediated by SA and JA (Irigoyen et al., 2020). PR proteins have been classified into 17 families. Among them, *PR-15* and *PR-16* families were only found in monocots. Although long-living trees would suffer more diverse set of fungal pathogens, the number of defense-related genes in poplars showed no different from *Arabidopsis* and rice, only *PR*-encoding chitinases and kunitz-type protease inhibitors are found to be more abundant in poplars (Kovalchuk et al., 2013). There are abundant *PR* genes in poplars, which are key for SA-mediated defense (Wei et al., 2020c). Several transcriptomic studies have found that *PR-1* expression is increased 100-fold higher than controls (Miranda et al., 2007; Azaiez et al., 2009; Boyle et al., 2010). In addition, the expression levels of *PR-2* (3-glucanase), *PR-3*, *PR-4* (chitinase), *PR-5* (thaumatin-like protein), *PR-6* (protease inhibitor), *PR-7* (l-aspartic acid protease) and *PR-9* (lignin peroxidase) are also increased after infection with pathogens (Azaiez et al., 2009; Duplessis et al., 2009). Interestingly, *PR-1* and *PR-2* genes induction are much larger in the susceptible genotype compared with the rust-resistant genotypes, indicating that the abundance of the rust pathogen determines the degree of *PR* gene induction in poplars (Ullah et al., 2022). Additionally, chitinase genes were upregulated in poplars after inoculation with *M. medusae* (Miranda et al., 2007) or *S. musiva* (Liang et al., 2014). Most of chitinase genes showed high transcript levels in early stage when infection with *Alternaria alternata* (Huang et al., 2022) (**Tables 1**, **2**). A chitinase gene (*Bbchit1*) from *Beauveria bassiana* was overexpressed in white poplar and enhanced resistance to a pathogenic fungus *C. chrysosperma* (Jia et al., 2010). Overexpression of *TLP* gene that belongs to *PR-5* family, could inhibit the growth of pathogens in poplars and enhance resistance to spots disease (**Table 2**) (Sun et al., 2020).

#### JA/ET-mediated signaling pathway against pathogens

Meanwhile, the JA/ET pathway is generally required for the predominant defense response against necrotrophic pathogens and herbivores (Yang et al., 2015). Genes involved in JA synthesis including *lipoxygenase* (*LOX*), *allene oxide synthase* (*AOS*), *allene oxide cyclase* (*AOC*), *12-oxophytodienoate reductase* (*OPR*) and *acyl-coenzyme oxidase* (*ACX*) were induced during biotrophic or necrotrophic pathogens infection (Azaiez et al., 2009; Huang et al., 2022). Interestingly, *LOX* can regulate the ROS accumulation in poplars to against pathogens (Huang et al., 2022). Genes involved in JA signal transduction pathways were also significantly induced, such as the MYC proteins, which are positive regulators in the JA signaling pathway, were upregulated during pathogen infection. While JAZ, which contains TIFY domains, a negative regulator of JA signal transduction (Pauwels and Goossens, 2011), was mainly downregulated during pathogen infection. The transcription factor TIFY has also been found to be a key element that contributes to phytohormone or stress responses. *TIFY* genes can be divided into four subfamilies, *TIFY*, *JAZ*, *ZML* and *PPD* (Xia et al., 2017). It has been reported that there are 24 *TIFY* genes in poplars (Xia et al., 2017). A study showed that most of the *TIFY* genes could respond to *M. larici-populina* infection, while genes expression patterns were different at different time points (Xia et al., 2017).

Furthermore, ET synthesis genes including *1-aminocyclopropane-1-carboxylate synthase* (*ACS*), and *1-aminocyclopropane-1-carboxylate oxidase* (*ACO*) were also induced after pathogens infection (Azaiez et al., 2009; Huang et al., 2022). ET activated *PtoRbohD/RbohF* expressions, which encode NADPH oxidases to induce H_2_O_2_ production in poplars to *Dothiorella gregaria* fungi (Liu et al., 2022). ET response factors (ERFs) bind to the ethylene-responsive element GCC-box, and the target genes are related to wounding and pathogen infection (Meng et al., 2013). A recent study showed that the transcript levels of 21 *ERF* genes were strikingly upregulated and 72 genes were downregulated in *P. nigra × P. deltoides* under *M. larici-populina* infection at 4 dpi, and the inactivation of *ERF* genes and disease resistance-related *ERF* target genes might result in poplar susceptibility to rust disease (Chen et al., 2019). Therefore, JA/ET pathway not only enhances necrotrophic fungus resistance on poplars, but also regulates the susceptibility of poplars to biotrophic fungus, which relate to the timing of the infection.

#### Crosstalk of SA and JA in poplar disease resistance

It is generally assumed that SA and JA are antagonistic in disease resistance in *Arabidopsis*, rice and tomato. Plants have to balance the costs and potential benefits of investing in defense to external stimulus (Pieterse et al., 2012; Thaler et al., 2012), but this antagonism is not obvious in poplar against rust disease. A study reported that both SA and JA contents were increased upon rust infection in black poplars, and transgenic poplar lines with high SA levels increased JA and flavonoid contents, and enhanced rust resistance (Ullah et al., 2019a; Ullah et al., 2022). Therefore, SA and JA pathways interact positively in poplars to decrease biotrophic pathogen growth (**Figure 2**). This difference may attribute to perennial woody plants can store large reserves of resources for defense, might have evolved SA- and JA-mediated co-defense systems without antagonism, while annual plants lack the defensive resources.

WRKY-TFs and *NONEXPRESSOR OF PR1* (*NPR1*) are known to be involved in modulating between SA- and JA-dependent responses in plants. There are 65 and 64 *PtrWRKY* gene promoters involved in SA and MeJA responses in poplars, respectively (Jiang et al., 2014). *WRKY70* activates the expression of *NPR1* and thus enhances *PR* expression, leading to the resistance of *Arabidopsis* to both biotrophic and hemibiotrophic pathogens but increasing plant susceptibility to necrotizing vegetative fungal pathogens (Li et al., 2004; Li et al., 2006; Shim et al., 2013). In poplars, *PsnWRKY70* enhance the resistance to *A. alternata* by activating genes involved in MAPK cascade and Ca^2+^ signaling, other members of *WRKYs*, and LRR domain proteins (Wang et al., 2022). *Arabidopsis* homolog transcripts WRKY70, WRKY51 and WRKY40 were strongly increased (more than 10-fold) in poplars after infection by *Melampsora* (Miranda et al., 2007; Azaiez et al., 2009). After exogenous SA treatment, nine genes in *PtrWRKYIII* were upregulated (*WRKY 89, WRKY 62, WRKY 64, WRKY63, WRKY41, WRKY55, WRKY-53, WRKY-54, WRKY-30*), while one gene (*PtrWRKY90*) was significantly downregulated in poplar (Wang et al., 2015). Particularly, *PtrWRKY89* induced by SA plays an important role in rust resistance by upregulating *PR* gene expression. *PtrWRKY18* and *PtrWRKY35* are potential target genes of *PtrWRKY89*, and they can increase resistance to *M. larici-populina* fungus (Jiang et al., 2017). Additionally, *PtrWRKY73* induced by SA in *P. tomentosa* could increase plant resistance to biotrophic pathogens but enhance sensitivity to the necrotrophic fungal pathogen (Duan et al., 2015). However, *PtrWRKY40* is similar with *AtWRKY40*, *AtWRKY18* and *AtWRKY60*, which had a side effect on the resistance of poplars to hemibiotrophic fungus (*D. gregaria*) by negatively regulating SA-related genes expression (Karim et al., 2015). To date, the role of *WRKY* genes has been explored extensively in poplars, which are associated with stress responses and phytohormones (Jiang et al., 2014).

Additionally, poplars can activate or inhibit the SA or JA pathway by proteins, such as *kunitz-type serine endopeptidase inhibitor* (*KTI*) regulation, which can restrict fungus growth by PCD (Chen et al., 2021). *KTI* controlled by the *cytochrome P450 family* (*CYP*). Thus, *CYP* genes have great effects on the JA and SA pathways (Xu et al., 2015a; Chen et al., 2021), while *KTI* and *CYP* genes were significantly induced by biotrophic fungus *Melampsora* (Miranda et al., 2007; Azaiez et al., 2009) and necrotrophic fungus *Sphaerulina* (Foster et al., 2015). In addition, plant defensins are antimicrobial peptides that represent a major barrier to invasion by pathogens. A study found overexpression of *PtDefensin* in poplars may change the crosstalk between the SA and JA signal pathways to increase the resistance to *S. populiperda* infection at the early stages (Wei et al., 2020a) (**Figure 2**). Similarly, *PtDefensin* overexpression transgenic poplars enhanced resistance to *S. populiperda* may be due to the upregulation of *PR1-1* and *MYC2-1* and downregulation of *JAZ1*, *COI1-1* and *COI1-2*, leading to activation of SA and JA signaling pathways. Host defense peptides (HDPs) are known as cationic antimicrobial peptides and almost found in all living organisms. MsrA2 peptide is proved to have the best antimicrobial potential among all HDPs, and overexpression of *MsrA2* in poplar leaves inhibited *S. musiva* growth (Yevtushenko and Misra, 2019). These findings suggest that antimicrobial peptides could be used for genetic engineering on woody plants to enhance disease resistance.

#### The role of ABA in fungal resistance

The role of abscisic acid (ABA) in disease resistance remains complex. ABA play positive role in plant immunity through stomatal closure and callose deposition during early stages of pathogen invasion, but may suppresses SA- or JA-dependent immunity in late disease resistance (Ton et al., 2009). ABA has been proven to regulate stomatal closure and rapidly accumulate ROS in stomatal cells (Kohler et al., 2003). It was indicated that exogenous ABA treatment increased poplar resistance against rust. Ullah et al. (2019a) subjected black poplars to drought stress followed by rust inoculation and found that endogenous ABA increased approximately 3-fold, and the growth of *M. larici-populina* significantly decreased by 10- and 6-fold at 4 and 8 dpi under drought stress, respectively. Due to the different invasion ways of biotrophic and necrotrophic fungus, ABA exerts greater influence on biotrophic fungi like rust which invaded through stomata (**Figure 2**). However, the mechanisms of ABA regulating poplars against pathogens in later stages of infection need to be explored.

#### Phytochemicals involved in defense responses

Several antimicrobial compounds are directly involved in the plant defense response to pathogens, including plant antibiotics, which are present in plants prior to infection, and plant antitoxins, which are produced in response to plant attack by pathogens (Silva et al., 2018). Many plants secondary metabolites are thought to serve as phytoalexins, such as flavan-3-ol (catechin, epicatechin) and phenolic acids that involved in phenylpropanoid pathway (Ullah et al., 2017; Ullah et al., 2019b). Phenols and flavonoids are vital products of the phenylpropanoid pathway, which plays important roles in plant disease resistance (Dixon et al., 2005; Syvertsen and Garcia-Sanchez, 2014; Dong and Lin, 2021). In poplars, it was reported that the accumulation of proanthocyanidin (PA) and flavan-3-ol in poplar leaves could inhibit rust hyphal growth and reduce rust colonization. The contents of catechin and PAs were strongly increased at 7 dpi, and their accumulation was significantly induced by SA (Ullah et al., 2017; Ullah et al., 2019a). In addition, moderately resistant poplars accumulate higher amounts of flavan-3-ols at the site of rust infection than susceptible poplars (Ullah et al., 2017). Additionally, the genes were significantly enriched in the flavonoid biosynthesis pathway in poplars after being infected by *M. brunnea* (*Marssonina*) (Zhang et al., 2018). The transcriptional responses of *P. trichocarpa × P. deltoides* to *M. medusae* showed that genes encoding enzymes of proanthocyanidin and flavonoid were strongly induced, such as *flavanone 3-hydroxylase* (*F3H*), *PAL*, *4CL*, *dihydroflavonol reductase* (*DFR*), *anthocyanidin reductase* (*ANR*) and *leucoanthocyanidin reductase* (*LAR*) (Miranda et al., 2007). Similarly, these genes were changed at different time points in poplars when attacked by *A. alternata*, but they were up-regulated at 2 dpi (Huang et al., 2022).

The MYB family is involved in the regulation of various physiological processes in plants. Flavonoid biosynthesis is regulated by the MYB family at the transcriptional level (Xu et al., 2015b). The activation of anthocyanin and PAs is different from that of other flavonoid branches because they need coactivators, *e.g*., basic-helix-loop-helix (bHLH) and WD40 (WDR), to interact with MYB to form MYB-bHLH-WD40 (MBW) (Ma and Constabel, 2019). SA can stimulate the expression of MBW that positively regulating the biosynthesis of anthocyanins and proanthocyanidins in poplars to reduce rust proliferation (Ullah et al., 2019a). In addition, *MYB134* (Mellway et al., 2009), *MYB119* (Cho et al., 2016) and *MYB115* (Wang et al., 2017a) are positive regulators to enhance resistance to fungus, while *MYB182* (Yoshida et al., 2015), *MYB57* (Wan et al., 2017), *MYB165* and *MYB192* (Ma et al., 2018) are negative regulators of proanthocyanins synthesis in poplars. However, *MYB6* can promote the biosynthesis of anthocyanins and proanthocyanins but suppress the formation of secondary cell walls in *P. euphratica* (Wang et al., 2019). *MYB118* (Wang et al., 2020), *MYB120* (Kim et al., 2021) and *MYB117* (Ma et al., 2021) are involved in anthocyanin and lignin biosynthesis, respectively. Additionally, MYB is widely involved in the phenylpropanoid pathway at the transcriptional level and can potentially precisely regulate lignin and flavonoid synthesis genes, which can enhance poplar disease resistance.

### Non-coding RNAs on poplars response to fungal pathogens

Most studies have focused on the function of protein-coding genes like *PR* in biotic stresses. However, large proportions of eukaryotic genomes are transcribed into RNAs that do not encode proteins. These transcripts are called noncoding RNAs (ncRNAs) and can be directly involved in the regulation of disease resistant genes (Li et al., 2021). ncRNAs are mainly classified into microRNAs (miRNAs), long ncRNAs (lncRNAs) and circular RNAs (circRNAs). Among them, miRNAs play important roles in disease resistance by cleaving target genes or repressing the translation of target mRNAs (Li et al., 2016). Li et al. (2016) studied the susceptibility of *P. nigra* × *P. deltoides* to *M. larici-populina*, they found that miRNAs could act directly or indirectly on disease-related genes or proteins. For example, CC-NBS-LRR class protein family, TIR-NBS-LRR class protein family, cellulose synthase genes and stress-inducible protein genes. However, none of them was responding to rust infection. Conversely, miRNAs related to PAMPs and PTIs were responsive to rust infection. Moreover, the miRNA-mediated posttranscriptional regulation of defense signaling genes was inactivated at the ETI and HR stages by infection with *M. larici-populina* (Li et al., 2016). After *P. trichocarpa* induced with canker pathogen (*Botryosphaeria dothidea*), 12 miRNAs were upregulated. Especially, miR156 responded to biotic and abiotic stresses in *Populus*, and miRNA-TF interaction networks in poplar canker were revealed. For example, miR159, miR164, and miR319 targeted MYB factors and MYB involved in their activation or repression, miR160-ARF (Auxin receptor factor) and miR167-ARF interaction were related to disease resistance (Zhao et al., 2012). Additionally, miRNAs also play crucial roles in the regulation of NBS-LRR and host defense responses (**Figure 1**). Liu et al. (2019) found *PsRPM1* and *PsRPS2/5*, which containing NBS-LRR domains, were significantly increased at later infection stages with rust, while miRNAs were down-regulated. These results indicated miRNAs were negatively regulated the expression of their target genes to enhance the resistance of poplars to rust fungus. Similarly, when poplars exposed to the hemibiotrophic fungus *C. gloeosporioides*, miR472a was down-regulated and NBS-LRRs were up-regulated, leading to a ROS burst and HR to against hemibiotrophic fungus. But when poplars are exposed to the necrotrophic fungus *Cytospora chrysosperma* miR472a negatively regulated *NBS-LRRs*, leading to PCD and resulting in necrotrophic fungus susceptibility (Su et al., 2018). Furthermore, plant lncRNAs might be a target of miRNAs and decrease the interaction between mRNAs and miRNAs by binding specific miRNAs. Moreover, lncRNAs that were located closed to protein-coding genes, were differentially expressed during pathogen infection (Wang et al., 2017b). Therefore, these results indicate that sRNAs play important roles in plant-pathogen interactions in poplars.

## Conclusion and prospects

Currently, the formation mechanism of plants induced disease resistance has been preliminarily revealed in some model species. However, the mechanisms of plants induced disease resistance are complex and may correspond with plant and pathogen species. Woody plants have more receptors and R proteins than herbs, providing them with better defenses strategies (Duplessis et al., 2009). When receptor proteins and R proteins sense the pathogen, then activate downstream signals. Different with *Arabidopsis*, SA and JA cooperated to against both biotrophic and necrotrophic fungus in poplar and the genes regulated SA signaling may be different from *Arabidopsis* (Ullah et al., 2019a; Ullah et al., 2022). Because perennial woody plants have higher resource utilization efficiency than annual plants, they generally use more resources for defense.

Although development and application of omics technologies have provided broadened insights into poplars defensive responses against fungal pathogens, there are still many gaps in our understanding of poplar defense against fungus. For instance, the mechanisms of diverse R proteins and receptors in poplar that sense different fungus are still unclear. Many plant-pathogen interaction candidate genes, such as *PRs*, *WRKYs*, *ERFs*, *TIFY*, *NDR1*, *EDS1* and *RISP*, which play important roles in poplar disease resistance, still need to be explored. In addition, plants defensive responses are complex, it is urgent to explore diverse crosstalk between different types of defensive responses in woody plants. The mechanism by which phytohormones interact with signaling molecules, *e.g*., ROS and NO, to influence poplar defense against pathogens also need to be investigated further. Further studies on the functions of ncRNAs in disease resistance and in regulating transcription and RNA silencing are of great significance.

## Author contributions

YZ collected the data and wrote the manuscript. HS, LX, and LY gave some advice. SZ designed the framework and revised the manuscript. All authors contributed to the article and approved the submitted version.

## Glossary

**Table:**

ABA	abscisic acid
ACS	ACC synthase gene
ACO	1-aminocyclopropane-1-carboxylate oxidase
ACX	acyl-coenzyme oxidase
ANR	anthocyanidin reductase
AOC	allene oxide cyclase gene
AOS	allene oxide synthase
AOX	mitochondrial alternative oxidase
APX	ascorbate peroxidase
Avr	avirulence gene
C4H	cinnamate 4-hydroxylase
CAD	cinnamyl alcohol dehydrogenase
CAT	catalase
CC	amino-terminalcoiled-coil
CDPKs	calcium-dependent protein kinase
CHI	chalconeisomerase
CHS	chalcone synthase
circRNAs	circular RNAs
CK	cytokinin
4CL	4-coumarate-CoA ligase
CsRFS	raffinose synthase gene
CYP	cytochrome P450 family
C3H	coumarate 3- hydroxylase
DFR	dihydroflavonol reductase
DIR	dirigent
EDS1	enhanced disease susceptibility 1
ERF	ethylene response factors
ET	ethylene
ETI	effector-triggered immunity
F3H	flavanone-3-hydroxylase
F5H	ferulate 5-hydroxylase
GOLS3	galactose synthase gene
GST	glutathione sulfur transferase
HCT	hydroxycinnamoyl transferase
HR	hypersensitive response
H_2_S	hydrogen sulfide
ICS	isochorismate synthase
IP3	inositol triphosphate
JA	jasmonic acid
JAZ	jasmonate-zim domain
KCS	3-ketoacyl CoA synthase
KTI	kunitz type serine endopeptidase inhibitor
LAR	leucoanthocyanidin reductase
lncRNAs	long ncRNAs
LOX	lipoxygenase
LRR-RLP	leucine-rich repeat receptor-like protein
MAPK	mitogen-activated protein kinase
MAPKKK5	mitogen activated protein kinase-kinase-kinase 5
miRNAs	microRNAs
NDR1	non-race-specific disease resistance 1
NLPs	necrosis and ethylene-inducing peptide 1-like proteins
NO	nitric oxide
ncRNAs	non-coding RNAs
NPR1	NONEXPRESSOR OF PR1
OPR	12-oxophytodienoate reductase
PAL	phenylalanine ammonia lyase
PAMPs	pathogen-associated molecular patterns
PAs	proanthocyanidins
PCD	programmed cell death
PIP5K	phosphatidylinositol 4-phosphate 5-kinase
POD	peroxidase
PR	pathogenesis-related gene
PRRs	pattern recognition receptors
PTI	pattern-triggered immunity
RISP	rust-induced secreted protein
RLKs	receptor-like kinases
RLP	Receptor-like protein
RNS	reactive nitrogen species
ROS	reactive oxygen species
SA	salicylic acid
SAR	systemic acquired resistance
SOD	superoxide dismutase
sRNA	small RNA
TAL	tyrosine ammonia lyase
TIR	toll-interleukin-1 receptor
